# Plastic Changes in Human Motor Cortical Output Induced by Random but not Closed-Loop Peripheral Stimulation: the Curse of Causality

**DOI:** 10.3389/fnhum.2016.00590

**Published:** 2016-11-15

**Authors:** Kenneth I. Brown, Elizabeth R. Williams, Felipe de Carvalho, Stuart N. Baker

**Affiliations:** Institute of Neuroscience, Newcastle UniversityNewcastle upon Tyne, UK

**Keywords:** motor cortex, plasticity, magnetic brain stimulation, closed loop, stimulation

## Abstract

Previous work showed that repetitive peripheral nerve stimulation can induce plastic changes in motor cortical output. Triggering electrical stimulation of central structures from natural activity can also generate plasticity. In this study, we tested whether triggering peripheral nerve stimulation from muscle activity would likewise induce changes in motor output. We developed a wearable electronic device capable of recording electromyogram (EMG) and delivering electrical stimulation under closed-loop control. This allowed paired stimuli to be delivered over longer periods than standard laboratory-based protocols. We tested this device in healthy human volunteers. Motor cortical output in relaxed thenar muscles was first assessed via the recruitment curve of responses to contralateral transcranial magnetic stimulation. The wearable device was then configured to record thenar EMG and stimulate the median nerve at the wrist (intensity around motor threshold, rate ~0.66 Hz). Subjects carried out normal daily activities for 4–7 h, before returning to the laboratory for repeated recruitment curve assessment. Four stimulation protocols were tested (9–14 subjects each): *No Stim*, no stimuli delivered; *Activity*, stimuli triggered by EMG activity above threshold; *Saved*, stimuli timed according to a previous *Activity* session in the same subject; *Rest*, stimuli given when EMG was silent. As expected, *No Stim* did not modify the recruitment curve. *Activity* and *Rest* conditions produced no significant effects across subjects, although there were changes in some individuals. *Saved* produced a significant and substantial increase, with average responses 2.14 times larger at 30% stimulator intensity above threshold. We argue that unavoidable delays in the closed loop feedback, due mainly to central and peripheral conduction times, mean that stimuli in the *Activity* paradigm arrived too late after cortical activation to generate consistent plastic changes. By contrast, stimuli delivered essentially at random during the *Saved* paradigm may have caused a generalized increase in cortical excitability akin to stochastic resonance, leading to plastic changes in corticospinal output. Our study demonstrates that non-invasive closed loop stimulation may be critically limited by conduction delays and the unavoidable constraint of causality.

## Introduction

Over the last two decades, several non-invasive stimulation protocols have been developed which can generate plastic changes in motor output. These include repetitive transcranial magnetic brain stimulation (rTMS, Pascual-Leone et al., 1994; Chen et al., 1997), transcranial direct current stimulation (tDCS, Nitsche and Paulus, 2000), paired associated stimulation (PAS, Stefan et al., 2000) and peripheral nerve stimulation (PNS, Ridding et al., 2000). The methods vary in whether they deliver a single stimulus (rTMS, tDCS, PNS) or two stimuli paired with an appropriate interval (PAS). Initial results from these approaches seem promising, and changes outlast the stimulation period by tens of minutes. However, typically measures eventually decline back to baseline levels. If these methods are to be of use as an intervention in clinical applications such as stroke rehabilitation, they need to be extended to yield more durable changes.

One solution to extending the duration of plastic changes might be to prolong the period over which stimuli are delivered. It is known that changes are only induced after around 45 min of afferent stimulation (McKay D. et al., 2002), and that repeating stimulation for 30 min per day for 3 days can lead to enhanced responses 1 week later (McKay D. R. et al., 2002). However, there are practical limits to delivery time for many plasticity protocols. Both rTMS and PAS rely on magnetic brain stimulation which requires a bulky machine and specialist operator, limiting application to the duration of a visit to the laboratory or clinic. tDCS operates with more portable equipment, which might allow use outside specialist settings (e.g., at home). However, there are no safety data on the effects of long-duration direct currents passed through the skin (Nitsche et al., 2003); electrodes are additionally bulky and unsightly (being placed on the scalp). It is thus probably infeasible to apply tDCS for more than a brief period each day, although repeated daily sessions do seem to enhance the effects (Boggio et al., 2007).

By contrast, PNS has many features making it suitable for prolonged use. There is no theoretical reason why electrical stimulation of a peripheral nerve over long periods should be harmful, and indeed in routine clinical use for pain management transcutaneous electrical nerve stimulation (TENS) has an excellent safety record (Simpson et al., 2014). Stimulation can be applied using portable battery-powered stimulators; electrodes are placed peripherally on the limbs, where they can be conveniently hidden from view under clothing, making the method more cosmetically acceptable. Stimulation can be directed to a given nerve, muscle or region of skin, allowing protocols to be designed which exploit the specific nature of the stimulus. Motor cortex (M1) receives extensive sensory inputs from both tactile (Rosén and Asanuma, 1972) and muscle afferents (Lemon and Porter, 1976; Cheney and Fetz, 1984), and motor cortical cells respond to peripheral nerve stimuli with short-latency discharge (Kozelj and Baker, 2014).

To date, two forms of PNS have been used to induce plastic changes in motor output. The simplest has directed stimulation to a single site. By varying stimulus intensity and frequency, it is possible to induce either facilitation or suppression in motor output (Chipchase et al., 2011). Alternatively, stimuli to two sites can be paired. The convergence of afferent volleys on a common central target then appears to generate an enhanced output. Stimulating both sites, but at separate times (non-associative stimulation), does not generate such effects (Ridding and Uy, 2003). This suggests that the rules governing plastic changes are similar to spike-timing dependent plasticity observed at the single synapse in animal preparations (Bi and Poo, 2001).

As an alternative to pairing two different stimuli, plastic changes can also be generated by linking one stimulus to the timing of naturally occurring activity. This has been demonstrated in animals by delivering weak intra-cortical or intra-spinal microstimulation triggered by spiking in a cortical neuron (Jackson et al., 2006; Nishimura et al., 2013), as well as in human subjects by timing TMS over the primary M1 to proceed a voluntary movement in response to a cue (Thabit et al., 2010). Plastic changes occur both when TMS is triggered by a voluntary movement, and when the movement is generated in response to a randomly-timed TMS discharge (Edwardson et al., 2014). To date no study has explored the potential of PNS triggered by voluntary activity to generate plastic changes in motor cortical output. In the field of stroke rehabilitation, however, functional electrical stimulation triggered by voluntary electromyogram (EMG) has been demonstrated to improve hand motor function on the affected side and to modify both spinal and cortical circuits (Bolton et al., 2004; Fujiwara et al., 2009). We expected that PNS triggered by voluntary EMG might also generate plastic changes in motor cortical output in healthy volunteers.

In this study, we developed a portable platform for investigating plasticity protocols, which we refer to as the “wearable electronic device”. This is capable of recording muscle activity (EMG), and delivering PNS to one or two nerves. A powerful on board processor allows real-time signal analysis, so that stimuli can be timed contingent on recorded activity in any way desired. We show that the device is capable of enhancing the motor output from M1 when worn for around 6 h whilst the subject carries out normal daily activities. Unexpectedly—at least for the stimulus site, intensity and rate tested here—plastic changes were greatest when stimuli were delivered randomly, rather than locked to periods of contraction or rest. We suggest that this may be due to conduction delays within the central and peripheral nervous systems, which limit induction of plasticity by activity-triggered stimulation.

## Materials and Methods

### Ethical Approval

Experiments were performed on 25 healthy human subjects with no history of neurological illness. Single subjects participated in between one and four of the different protocols described below (mean 1.9 protocols/subject). All procedures were approved by the ethics committee of Newcastle University Medical Faculty; subjects provided informed written consent in accordance with the Declaration of Helsinki.

### Transcranial Magnetic Stimulation Recruitment Curve

Motor cortical output was assessed by compiling a response recruitment curve using transcranial magnetic stimulation of the contralateral M1. Surface EMG was recorded from the thenar muscles of the right hand, using adhesive electrodes (Kendall H34SG Foam Hydrogel, 50 mm × 45 mm, MedCat Supplies, Holland). Signals were amplified (band pass 30 Hz–2 kHz, gain 500–5000) and captured to hard disc at 5000 samples/s using a micro1401 interface (Cambridge Electronic Design Ltd, Cambridge, UK).

Subjects were seated comfortably in front of a table, with their head resting in a holder which supported the chin and forehead. TMS was delivered using a Magstim 200 stimulator (The Magstim Company Ltd, Whitland, UK) and figure-of-eight coil (diameter of each coil 70 mm). The subject wore on a headband the transmitter for a magnetic-field based 3D positioning system (Polhemus Liberty 240/8); the sensor for the system was securely fixed to the coil handle. The coil was first positioned at the optimal location to elicit a motor evoked potential (MEP) in the thenar EMG, with the coil held at an angle of 45° to the midline, handle pointing posteriorly. This location was then stored by a custom program which read the Polhemus sensor position. All subsequent TMS was delivered at this location and coil orientation, verified by using continuous feedback of errors in position and angle on a computer screen viewed by the experimenter. With the subject relaxed, an estimate of the threshold was obtained as the intensity which yielded responses in 50% of trials. The lowest intensity tested was set just below this value (rounded down to nearest 5% of maximum stimulator output, MSO). Responses to ten stimulus intensities were then tested, ranging from this value upwards in equal increments of 5% MSO. Stimulus intensities were interleaved in pseudo-random order, with 10 stimuli at each intensity (0.2 Hz rate).

At the end of this recording, the location of the position transmitter and its headband was marked on the forehead using “invisible ink” which fluoresced under weak ultraviolet illumination. The headband was then removed.

### Wearable Electronic Device

The custom device used in this study was based around a dsPIC33 microcontroller (Microchip Inc, Chandler, AZ, USA) running at 40 million instructions per second. The device had two amplifiers suitable for EMG recording (band pass 30 Hz–2 kHz, gain digitally adjustable 198–2239×); amplified EMG was sampled by the analog-to-digital convertor of the microcontroller with 12 bit resolution at 1 k samples/s/channel (3.3V full scale deflection). Note that ideally with this sampling rate, a 500 Hz low pass filter should have been used (the Nyquist limit). However, we verified with separate recordings at higher sampling rate that typically around 0.5% of the total power of a surface EMG recording lies in the 500–2000 Hz range; any aliasing would therefore have been negligible. Two nerve stimulators provided isolated constant-current stimuli, with currents up to 20 mA, and 220 V compliance. The current was adjusted manually using a miniature potentiometer; pulse width could be digitally controlled, but was fixed here at 150 μs. The stimulators could be powered down when not in use, which reduced system power consumption and extended battery life. After turning on, there was a 10 ms delay before the stimulators became able to deliver a stimulus. Data storage used secure digital (SD) card flash memory, formatted using the FAT32 system allowing files to be downloaded easily to a personal computer fitted with SD card reader. The user interface incorporated a six line 36 mm × 26 mm liquid crystal display (LCD), which could show graphical plots (e.g., EMG traces) as well as text. A four-direction joystick allowed navigation of menus for configuration. The device measured 143 mm × 68 mm × 25 mm; power was supplied by three AA alkaline batteries, which gave a runtime of around 10 h. Programs running on the device were written in C, using the MPLAB development environment and cross-compilers (Microchip Inc., Chandler, AZ, USA) running on a standard Windows-based PC.

### General Experimental Protocol

**Figure 1:**
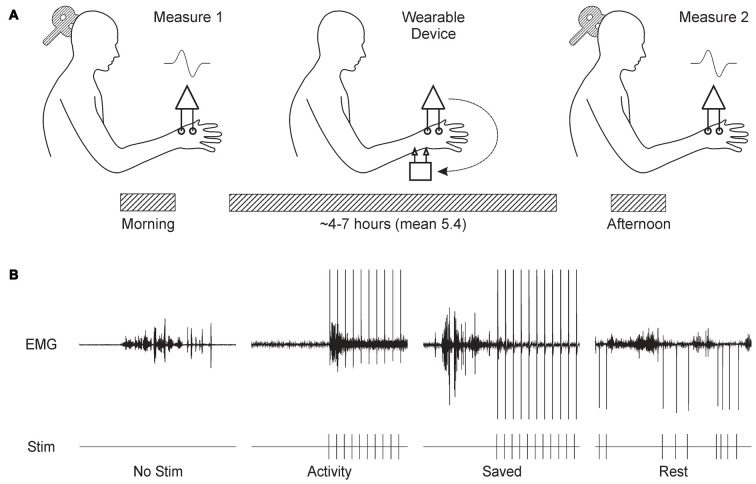
**Overview of experimental design. (A)** Subjects visited the laboratory in the morning, and the recruitment curve for motor evoked potentials (MEPs) from the thenar muscles following transcranial magnetic brain stimulation (TMS) over contralateral motor cortex (M1) was recorded. Subjects were fitted with the wearable electronic device, which delivered stimulation to the median nerve at the wrist whilst recording thenar electromyogram (EMG). The subject then left the laboratory, and carried out usual daily activities. Around 6 h later, the subject returned to the laboratory for a repeat measurement of the MEP recruitment curve. **(B)** Illustration of the stimulus protocols tested. Each panel shows a brief section of EMG recordings made by the wearable device above markers indicating the times of stimulus delivery. *No Stim*, no stimuli were given. *Activity*, stimuli were given when smoothed rectified EMG rose above a threshold level. *Saved*, stimulus timing was determined from a previous *Activity* protocol in the same subject. *Rest*, stimuli were delivered after EMG had been below a threshold level for 300 ms.

Figure 1A shows schematically the basic experimental paradigm. The experiment began around 9 am with a measurement of MEPs from the thenar muscles of the right hand. MEPs were recorded at increasing stimulus intensities, allowing construction of a response recruitment curve. The thenar EMG electrodes were then disconnected from the lab-based amplifiers, and connected to the wearable device. Similar surface electrodes placed over the median nerve at the wrist were connected to the device’s stimulator output (cathode proximal). The device was first placed in display mode, where EMG traces were shown on the built-in LCD display. Whilst the subject made strong contractions, EMG gain was adjusted to ensure that the signal did not clip on the input to the analog-to-digital convertor. Secondly, the device was configured to deliver median nerve stimuli at 1 Hz; the intensity was increased until small thenar twitches were just visible. The device does not provide a readout of current intensity in milliamps, but this is not especially meaningful as it is greatly affected by factors such as amount of sub-cutaneous fat, arm size and exact electrode placement. By working consistently at motor threshold, we ensured that effective stimulation intensities were comparable between subjects. Finally, a ~30 s recording of EMG was made during a series of contractions, followed by a period of relaxation. This data file was downloaded from the device immediately, and analyzed to choose thresholds for “rest” and “active” periods based on a cumulative distribution histogram of the rectified and smoothed EMG. Smoothing used a 5 Hz low-pass causal filter (order 40), and an identical filtering algorithm as that which would run on the device. These thresholds were coded into the program which would implement the real-time stimulus paradigm, and downloaded to the device.

The device was then set to run the stimulus paradigm. Subjects chose either to wear the device in a pouch attached to a belt around the waist, or to place it in a pocket. Cables were routed under/over clothing according to subject preference to cause minimal interference with normal movement. The subject then left the laboratory, and carried out their normal daily routine. Since many of our subjects were staff or students in the university, this involved a wide range of work, including both desk-based computer use and practical laboratory tasks (e.g., soldering). Subjects tolerated the device and electrodes well, often reporting that they quickly ceased to notice the stimuli.

Between 4 h and 7 h later, subjects returned to the laboratory. The wearable device paradigm was stopped, and the thenar EMG electrodes were reconnected to the laboratory amplifiers. The headband with position measurement transmitter was replaced on the forehead, guided by the fluorescent ink marks made earlier. A repeated measurement of the TMS recruitment curve was then made. Data files containing EMG activity and stimulus times were downloaded from the wearable device.

We tested four different paradigms, as illustrated in Figure 1B and described below:

*No Stimulus* Subjects wore the device, but no median nerve stimuli were given.

*Activity* Median nerve stimulation was triggered 10 ms after the rectified and smoothed EMG recording exceeded a threshold indicating muscle activity. The 10 ms delay was an unavoidable feature of our system, relating to the need to power up the stimulator first. After each stimulus, there was a 750 ms “dead time” during which no further stimulation could occur.

*Saved* Stimulus timing was dictated by that given to this subject in a previous occasion when running the *Activity* paradigm. Hence, although exactly the same sequence of inter-stimulus intervals was used as before, this bore no relation to the current EMG activity.

*Rest* Stimuli were delivered only when the EMG had been below the rest threshold for more than 300 ms. As for *Activity*, each stimulus was followed by a dead time. This began at 1507 ms, which corresponded to the mean inter-stimulus interval across all subjects in the *Activity* paradigm. After each stimulus, the mean inter-stimulus interval for that session up to that point was calculated. If it was larger than 1507 ms, the dead time was reduced by 12.5%; if the mean interval was smaller than 1507 ms, the dead time was increased by 12.5%. The dead time was not allowed to fall below 750 ms. This iterative adjustment ensured that the mean inter-stimulus interval in the *Rest* paradigm was close to that in *Activity*.

In many cases, the same subject took part in experiments testing several different paradigms; studies in the same subject were always separated by more than 1 week.

### Analysis

The peak-to-peak height of each MEP was measured individually following each stimulus; the mean and SEM for each intensity were determined, and plotted vs. intensity to yield a recruitment curve. To average recruitment curves across subjects, intensities were normalized by subtracting the lowest intensity tested which gave a significant response. Response size was expressed as a percentage of the largest average response seen in each subject in the recordings made at the start of that day. Data files recorded by the wearable device were analyzed only to measure the number of median nerve stimuli delivered, and the duration of the paradigm.

Testing for significant changes poses particular challenges in a study of plasticity, since it is known that subjects may show widely different responses to the same protocol (Wiethoff et al., 2014). To avoid missing effects which occurred in only some subjects, or had opposite directions in different subjects, we first tested for significant response changes within each subject and intensity, using paired *t*-tests and a threshold of *P* < 0.05. We then counted the number of significant changes seen across the subject population at a given intensity. If we have N subjects, then we can compute the probability of seeing M or more significant differences from the binomial distribution with *P*(hit) = 0.05:

(1) $$\text{P}(\text{M}\;\text{or}\;\text{more}\;\text{changes}\;\text{out}\;\text{of}\;\text{N})\;\text{=​​}\sum_{\text{m}\;\text{=}\;\text{M}}^{\text{N}}\text{​}\frac{\text{m}!(\text{N-m})!}{\text{N}!}\text{0}.{\text{05}}^{\text{m}}\text{0}.{\text{95}}^{\text{N-m}}$$

At a given intensity, if this probability was less than 0.05, we accepted that a significant (although possibly heterogeneous) effect had occurred across the population. We have used similar binomial statistics in the past to check for the overall significance of an effect when multiple bins cross a significance threshold in coherence analysis (Witham et al., 2010).

A similar approach was used to correct for multiple comparisons consequent on recording at 10 intensities. We measured the proportion of intensities which showed significant changes (*P* < 0.05), and then computed the probability of seeing this many or more using the binomial distribution.

All analysis was carried out using custom scripts in the MATLAB environment (Mathworks Inc., Natick, MA, USA).

## Results

**Figure 2:**
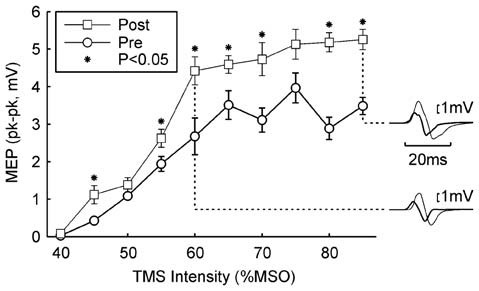
**Example result in one subject.** The peak-to-peak height of the MEP is plotted against the TMS intensity (mean ± SEM). Circles show responses recorded in the morning, before wearable device use; squares show responses recorded in the afternoon, after 5.4 h of wearable device action. Asterisks denote responses which were significantly different between the two measurements (*P* < 0.05). Insets on the right illustrate averaged MEPs for intensities of 60% and 85% maximum stimulator output (MSO); thick traces are MEPs before wearable device use, thin lines after. Protocol *Rest*.

Figure 2 shows a typical result from a single subject from the *Rest* paradigm. Following 5.5 h of the wearable device stimulation (during which time 12,512 stimuli were given), the MEP recruitment curve was shifted upwards, with significant differences seen at 7/10 intensities (*P* < 0.05, paired *t*-test; overall significance of this result *P* < 10^−7^). Inset traces to Figure 2 show examples of MEPs before and after the wearable device stimulation for two example intensities.

**Figure 3:**
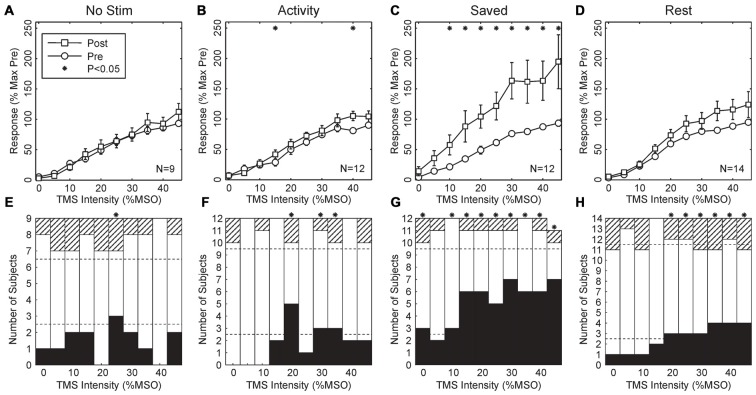
**Population results across all subjects. (A–D)** Averaged MEP recruitment curves for the conditions indicated above each plot. Circles indicate curves measured in the morning before wearable device use, squares indicate curves measured in the afternoon after the device had been worn. Each point is the mean ± SEM calculated across subjects. Intensities have been normalized by subtracting the first intensity with a response significantly different from zero in each subject. Responses are expressed as a percentage of the maximum response seen in the morning recordings for each subject. Subject numbers (N) are shown inset to each plot. **(E–H)** Bar charts showing the number of subjects with significant changes in their recruitment curves at each intensity. Black bars mark subjects with significant increases after wearable device use; hatched bars show subjects with a significant decrease; white bars, no change. Dotted lines indicate thresholds, based on the binomial distribution. On the null hypothesis, both black and hatched bars should remain within these lines. Asterisks throughout mark results with significant changes before and after wearable device use (*P* < 0.05).

Figure 3 shows results from the four paradigms tested. In Figures 3A–D, recruitment curves have been averaged across all subjects. As expected, there was no change for the *No stimulus* condition. This is an important control, demonstrating that, for example, the approach used to maintain the same stimulus coil location before and after the wearable device session was effective, and also that there were no systematic changes in recruitment curve associated with the different time of day of the two recording sessions (morning vs. late afternoon).

Surprisingly, when median nerve stimuli were triggered by activity in thenar EMG, there was also no overall significant change in the recruitment curve (Figure 3B, *Activity*; 2/10 points changed at *P* < 0.05, overall *P* = 0.086, binomial test). However, when the timing of stimuli delivered during the *Activity* paradigm were repeated on another day (Figure 3C, *Saved*), a substantial upwards shift in the recruitment curve was seen (8/10 points different *P* < 0.05 *t*-test; overall *P* = 1.6 × 10^−9^). Finally, when stimuli were triggered from a period of EMG inactivity (Figure 3D, *Rest*), although there appeared to be a small shift upwards in the recruitment curve, this did not reach significance at any intensity.

Figures 3A–D indicates the average recruitment curve changes seen across all subjects. Whilst this is a convenient representation of the population result, it may mask substantial heterogeneity across individual subjects. Accordingly, Figures 3E–H shows the proportion of changes which were significant in individual subjects, with black bars indicating increased responses following the wearable device paradigm, and hatched bars indicating decreased responses. In agreement with the across-subject averages, there was little consistent change in either the *No Stimulus* or *Activity* conditions, although for 3/10 intensities there were significantly more increased responses than expected by chance, a result which was just significant (*P* = 0.011 from binomial distribution). For the *Saved* paradigm, around half of the subjects showed significant increases at higher intensities, and for 9/10 intensities there were more subjects showing significant changes than expected by chance (*P* = 1.8 × 10^−11^).

For the *Rest* paradigm, Figure 3D indicated that no significant changes occurred on average across the population. However, Figure 3H reveals that for 6/10 intensities, significant changes occurred in more subjects than expected by chance, but that this often comprised changes in opposite directions for different subjects. For example, at the highest intensity tested 4/14 subjects showed significantly increased MEPs, and 3/14 significantly decreased MEPs. To see such changes in 7/14 subjects cannot be dismissed as chance (*P* = 2.0 × 10^−6^, binomial distribution); this must reflect a genuine heterogeneity across the subject population.

One possible source of the variability in effects between subjects could be differences in the time that the wearable device was applied. We checked for this in the Saved condition by assessing the correlation between either duration that the wearable device was applied, or the number of stimuli which it delivered, and the percentage change in MEP seen in each subject at 40% above threshold. In neither case was the correlation significant (*P* > 0.05).

The *Saved* condition was intended as a control, as we expected that muscle activity during this condition would not bear any correlation with the activity previously recorded in the *Activity* condition and used to trigger the stimuli. We verified that this was the case by computing the cross-correlation (Kilner et al., 2002) between the rectified EMG recorded by the wearable device in *Saved* and *Activity* for the same subject, for lags up to 1 s. A period of 100 ms after each stimulus was set to zero in each recording, to avoid artifactual correlations generated by the stimulus artifact or any motor response to the stimulus (whether directly evoked, or voluntary). In no case did the correlation coefficient *r*^2^ at any lag exceed 3 × 10^−4^, confirming that muscle activity in the *Saved* condition was indeed unrelated to that from the previous *Activity* recording.

**Figure 4:**
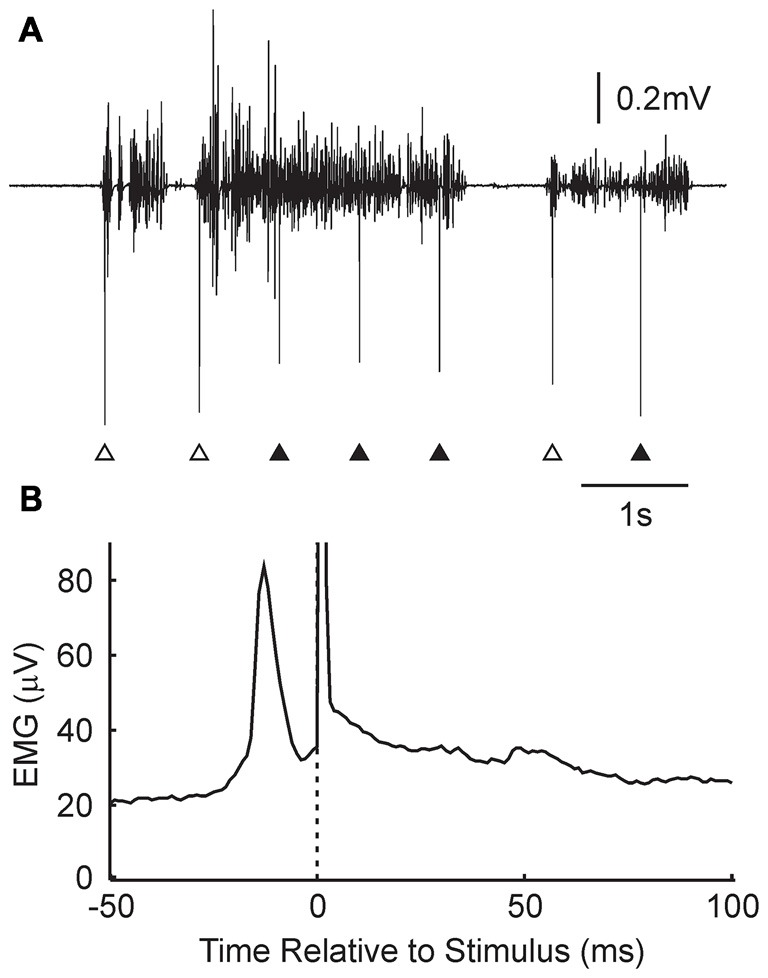
**Details of stimulus timing in the *Activity* condition. (A)** Example EMG recording from the wearable device, and associated stimulus times (arrowheads). Some stimuli were given just after the onset of a contraction (open arrowheads); others occurred in the middle of a tonic contraction (filled arrowheads). **(B)** Stimulus triggered average of rectified EMG, for the subject shown in **(A).** A narrow peak in activity is visible just prior to the stimulus (time zero, marked by dotted line and stimulus artifact). *N* = 11,024 stimuli.

Further insight into why changes were not produced consistently in the *Activity* condition can be gained by examining the detailed timing of the stimuli relative to EMG. Figure 4A shows a brief section of EMG recording from one subject. Over this time, there were three periods of EMG activity, separated by regions of EMG silence. Stimuli were delivered, according to the algorithm, whenever activity was detected, subject to an imposed dead time between stimuli of 750 ms. This meant that the three stimuli marked with open arrowheads were delivered just after the onset of the contraction, whereas those indicated by closed arrowheads were delivered in the midst of a reasonably consistent period of activity.

Figure 4B presents a stimulus-triggered average of the EMG, compiled from all 11,024 stimuli delivered over the 5.4 h that the device was worn by this subject. There was a sharp peak in activity which began at 22 ms and peaked at 13 ms before the stimulus was delivered.

## Discussion

In this study, we show that a wearable electronic device can induce plastic changes in motor cortical output. The portable nature of the stimulation allowed us to explore a different region of stimulus parameters from those previously used in PNS plasticity paradigms. We used a stimulus intensity which was close to motor threshold, and a stimulus rate around 0.66 Hz, which made the stimulus comfortable to the subject for long term use. Previous work has typically required much higher rates around 10 Hz, and intensities which gave visible muscle twitches (Ridding et al., 2000, 2001; Charlton et al., 2003). Importantly, we found differences in the ability to induce plastic changes depending on how stimuli were timed. Given previous work, we expected that pairing stimuli with periods of muscle activity would produce greater changes than constant stimulation. However, this was not the case, and the largest effects were produced when stimuli were instead given at random relative to contraction.

PNS will activate a mixture of peripheral axons, which will have effects on a variety of central circuits. One previous study used PNS above motor threshold, and found effects which could be best explained by antidromic stimulation of the motor axons (Taylor and Martin, 2009). However, in the present work we stimulated close to motor threshold, so that few, if any motor axons in the median nerve would have been activated; effects were thus probably produced by afferents. At these intensities, axons innervating group I muscle receptors would certainly have been activated (Jack, 1978), although there would also be some stimulation of cutaneous fibers. The two most likely sites where afferent activity could generate plastic changes leading to modified responses to TMS are in the spinal cord, and M1; we cannot further distinguish these possibilities in the present study.

### Mechanisms of Plastic Changes

**Figure 5:**
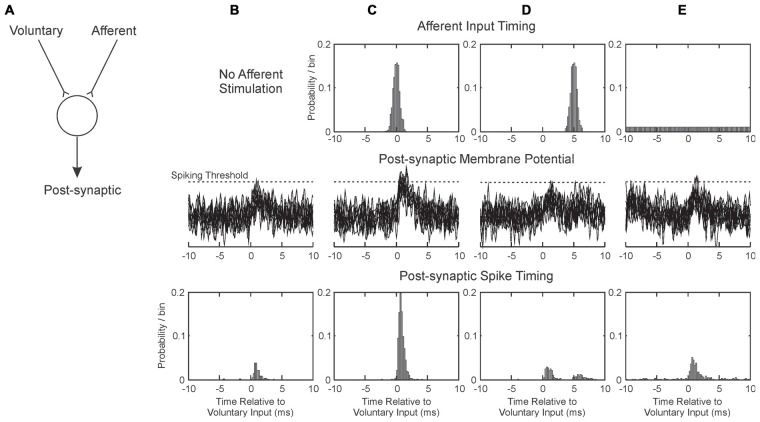
**Illustration of how pairing afferent stimulation with voluntary drive may produce conditions suitable for plasticity. (A)** We consider a single post-synaptic cell receiving excitatory synaptic inputs from two sources: one activated by voluntary motor commands, and one from afferent input. These sum at the cell membrane; if they exceed spiking threshold, the post-synaptic cell generates a spike. Each column **(B–E)** illustrates a different situation of afferent activation. The three rows represent respectively the timing of the afferent input relative to the voluntary input (at time zero), overlain example traces of the post-synaptic membrane potential and the timing of the discharge of the post-synaptic cell. **(B)** Without afferent input. **(C)** Afferent input activated close in time to voluntary input. **(D)** Afferent input activated on average 5 ms after voluntary input. **(E)** Afferent input activated at random relative to the voluntary input. EPSPs were simulated as alpha functions, with rise time 1 ms. Amplitudes in arbitrary units were 0.6 for Voluntary and 0.5 for Afferent inputs; spike threshold was at 1.2. Post-synaptic cell membrane noise was modeled as Gaussian noise bandlimited to 2 kHz, standard deviation 0.3.

Figure 5 illustrates, in a simple simulation, how various protocols for pairing afferent activation with voluntary drive might induce plastic changes. Assume that two sources of input converge onto a common post-synaptic cell: one is activated by voluntary drive (e.g., from higher motor areas), and the other by afferent stimulation (Figure 5A). Further assume that the synapse conveying voluntary activity is susceptible to spike-timing dependent plasticity (Bi and Poo, 2001), which has been demonstrated in human M1 (Wolters et al., 2003). EPSPs sum linearly with band-limited noise, and that the cell responds with a spike whenever its membrane potential crosses a threshold.

When the afferent input is not stimulated (Figure 5B), the small EPSP generated by the voluntary input only occasionally crosses threshold (Figure 5B, middle row), generating spikes immediately after the pre-synaptic voluntary input spike (Figure 5B, bottom row). With the parameters chosen (see figure legend), a post-synaptic response is generated after only 17% of voluntary inputs. We assume that in this baseline condition, any potentiation of the synapse by this “pre-before-post” activity is canceled out by other random spiking, and no change in synaptic strength occurs. Figure 5C illustrates the condition when the afferent input is timed to coincide closely with the voluntary. The probability of threshold crossing greatly increases: now 90% of voluntary inputs generate a post-synaptic spike. This close and reliable time locking will generate potentiation of the voluntary input (Markram et al., 1997).

Figure 5D shows how the situation changes when the afferent input is slightly delayed relative to the voluntary input. Now there is no coincidence of EPSPs; the afferent input produces a second peak in the post-synaptic spike time histogram (Figure 5D, bottom row), but even summing together the first and second peaks a response follows only 26% of the voluntary inputs. This will produce only slight potentiation, not only because of the low response probability, but also because the spikes in the second peak occur with some delay after the pre-synaptic input. The greater the delay from pre- to post-synaptic activation, the less potentiation will occur (Markram et al., 1997).

In the *Activity* condition of our experiments, many stimuli were delivered around 20 ms after the onset of a contraction (Figure 4). Further delays will be added by the conduction time from M1 to hand muscle (at least 20 ms in human, although possibly substantially longer, Witham et al., 2010), and a similar afferent conduction time for the response to the stimulus to reach the cortex. Overall, stimulus-evoked activity will come to M1 >60 ms after the voluntary activity which triggered it. Shorter, but still substantial, delays would pertain for the spinal cord. There is no chance of generating input coincidence as in Figure 5C; rather the afferent input will produce a weak, delayed peak analogous to Figure 5D. This is unlikely to generate plastic changes.

Figure 5E shows the situation where afferent input is activated at random relative to the voluntary input. This has the effect of increasing post-synaptic membrane noise, and increasing the probability of threshold crossings following the voluntary EPSP to 38%. The delay after the pre-synaptic spike is short, and should lead to synaptic potentiation. This mechanism may explain why the most impressive increases in corticospinal output were produced by the *Saved* condition: the randomly timed stimuli may have caused a general increase in background excitability. The phenomenon may be comparable to that of stochastic resonance, in which addition of a small amount of noise to a system improves its performance (see for example Trenado et al., 2014).

In the *Rest* condition, two effects may interact. Stimuli occurred if the EMG was below a threshold for >300 ms. This meant that many stimuli were triggered just after a contraction, soon after many M1 neurons ceased firing. This would tend to produce synaptic depression. By contrast, during long rest periods stimuli were delivered quasi randomly relative to baseline spiking (see Figure 1), which may produce synaptic potentiation by analogy with Figure 5E. In our experimental conditions, the net result for this paradigm seemed to be a small facilitation (Figures 3D,H), although there was considerable heterogeneity across subjects.

The parameters used to generate Figure 5 have been deliberately chosen to make a point, and little should be read into the detailed numbers. This illustration considers only one post-synaptic cell, and a single spike response to voluntary input. In reality, a whole population of neurons may be affected by plastic processes. Cells fire spontaneously, which will dilute the induction of plasticity by adding many randomly timed spikes. However, Figure 5 does perhaps provide a framework to explain the results that we obtained.

The inability of triggered stimulation to generate plastic changes is an unavoidable feature of a causal system, which cannot avoid a delay between the triggering neural activity and the stimulus-evoked activity reaching the target. Previous work which succeeded in generating plastic changes used configurations where this delay was minimized. Thabit et al. (2010) delivered TMS to M1 at a defined interval after the cue in a reaction time task. In that case, it was possible to predict the time of contraction from the cue, and hence ensure that stimuli were given to coincide with cortical activity (see Figure 5C). Edwardson et al. (2014) delivered TMS to M1 triggered by a muscle contraction, and, working in monkey, Lucas and Fetz (2013) gave weak intracortical microstimuli to M1 triggered by EMG. In both cases plastic changes in cortical output were produced. By delivering the stimuli direct to the cortex these studies avoided the afferent conduction delay. Additionally, in monkey the corticomuscular conduction delay is around half that in human (~10 ms to hand muscles). These studies were therefore probably working in the regime illustrated by Figure 5D: responses to the stimulus were delayed relative to the triggering cortical activity, but with sufficiently small intervals that plastic changes could still be induced.

Two further closed loop stimulation studies used single neuron spiking activity from M1 to trigger stimulation of either another site in M1 (Jackson et al., 2006) or the spinal cord (Nishimura et al., 2013). In each case, the spikes were detected at the cell soma, but activity would arrive at the stimulated site later due to cortico-cortical or corticospinal conduction delays. The stimulus-locked input would occur simultaneously with arrival of the endogenous activity at the target site, giving conditions close to those illustrated in Figure 5C. It is thus unsurprising that robust plastic changes were induced in those studies.

Two further potential confounding factors must be considered. It is possible that the surface electrodes used to deliver the median nerve stimulation were displaced during voluntary movement, so that fewer fibers were activated in the *Activity* condition compared with *Rest* or *Saved*. This cannot be ruled out, but it is unlikely to have been significant. Stimuli were delivered close to motor threshold. Afferents are activated sufficiently to detect physiological effects in human subjects at intensities as low as 40% of motor threshold (Malmgren and Pierrot-Deseilligny, 1988). Although the afferent volley may have been modulated slightly by electrode displacement, it would have been substantial in all conditions tested and should still have been sufficient to induce plastic changes. Secondly, because *Saved* necessarily followed a previous *Activity* session, there could be an order effect whereby significant changes would only occur on the second day of wearable device stimulation. This is however unlikely, as at least 1 week separated the two sessions.

### Variability Across Subjects

Electrophysiological studies in human subjects often report average effects, combined across all participants. Previous work has revealed that there is substantial heterogeneity in the responses of individual subjects to plasticity protocols (Charlton et al., 2003). This may be influenced by genetic factors (Cheeran et al., 2008), day-to-day fluctuations in the physiology of circadian rhythms (Clow et al., 2014) or individual differences in how non-invasive stimuli activate the brain (Wiethoff et al., 2014). In this study, we found a clear facilitation of motor output at the population level using stimuli applied at random relative to voluntary activity (Figure 4C). A similar result to the population effect was statistically significant at the individual level in around half of the subjects (Figure 4G). This may partly be statistical thresholding—the effects in the other subjects may have simply been too small to be detected—but it is also likely to reflect a genuine heterogeneity in response.

Many factors may influence the consequence of a given protocol on motor output. These include neural firing rates in the centers which are affected, the precise shape of the STDP curve (Feldman, 2012), and the profiles of rate modulation which occur during activity and rest. As well as individual differences in basic physiological processes, these are also likely to be influenced in our protocol by the activity which the subject carries out whilst fitted with the wearable device. Further improvements in these protocols to generate more robust changes in all subjects is likely to require a better understanding, at the synaptic and cellular level, of the mechanisms by which motor output is modified.

## Author Contributions

SNB designed the study and obtained funding. FC designed and built the wearable electronic device. KIB, ERW and SNB performed the experiments in SNB’s laboratory in Newcastle and performed the data analysis. SNB wrote the first draft of the manuscript. All authors contributed to revisions of the manuscript and approved the final version.

## Funding

This work was funded by the Medical Research Council Grant no. (G0801705) and the Wellcome Trust Grant no. (101002).

## Conflict of Interest Statement

The authors declare that the research was conducted in the absence of any commercial or financial relationships that could be construed as a potential conflict of interest.
